# Prevalence of Anemia among Chinese Rural Residents

**DOI:** 10.3390/nu9030192

**Published:** 2017-02-24

**Authors:** Min Li, Yichun Hu, Deqian Mao, Rui Wang, Jing Chen, Weidong Li, Xiaoguang Yang, Jianhua Piao, Lichen Yang

**Affiliations:** The Key Laboratory of Trace Element Nutrition MOH, National Institute for Nutrition and Health, Chinese Center for Disease Control and Prevention, Room 103, 29 Nan Wei Road, Xuanwu District, Beijing 100050, China; lmlovely@163.com (M.L.); huyichun0319@163.com (Y.H.); dqmao@126.com (D.M.); bluingwang@gmail.com (R.W.); chenjing1909@sina.com (J.C.); weidongli0507@126.com (W.L.); xgyangcdc@vip.sina.com (X.Y.); piao_jianhua@sohu.com (J.P.)

**Keywords:** anemia, Chinese rural residents, hemoglobin, nutrition survey

## Abstract

This paper presents an analysis of the level of blood hemoglobin and the rates of anemia in Chinese rural residents in the 2010–2012 National Nutrition and Health Survey, and the change in its prevalence in rural residents during the last ten years. Our methodology included data from the Chinese Nutrition and Health Surveillance in 2010–2012, where samples were selected through the method of probability proportion to size. The study objects were from 150 sites in provinces, autonomous regions, or municipalities in China. The concentration of blood hemoglobin was determined using the cyanmethemoglobin method. Anemia was judged by the anemia standard recommended by the World Health Organization (WHO), combined with elevation correction standard. The level of blood hemoglobin, the prevalence of anemia, and the 95% CI (Confidence interval) value were analyzed using complex sampling weighted processing, combined with the population figures released by the National Bureau of Statistics in 2009. Our results indicate that the level of blood hemoglobin of the Chinese rural area population was 145.92 ± 0.83 g/L, with the prevalence of anemia in the Chinese rural population at 9.7% (95% CI: 9.4%–10.0%). The prevalence of anemia in children 6–11 years old was 5.5% (95% CI: 5.0%–6.0%), 8.1% (95% CI: 7.5%–8.7%) for 12–17-year-old teenagers, 10.0% (95% CI: 9.4%–10.6%) for 18–44-year-old adults, 9.6% (95% CI: 9.0%–10.1%) for 45–59-year-old adults, and 12.6% (95% CI: 11.9%–13.3%) for the elderly above 60 years old. Our conclusion shows that the prevalence of anemia in the Chinese rural population in 2010–2012 had obviously decreased compared to the last decade; however, women of reproductive age and the elderly still had a high prevalence of anemia.

## 1. Introduction

Anemia is a condition in which the number and size of red blood cells, or the hemoglobin concentration, falls below an established cut-off value, consequently impairing the capacity of the blood to transport oxygen around the body. It is a common nutritional problem worldwide, and more than a quarter of the global population—especially in developing countries—suffer from anemia [1]. Anemia is common in infants and young children, women of childbearing age, pregnant women, lactating women, and the elderly. There are many reasons that can lead to anemia, such as iron deficiency, a lack of vitamin B12, sickle cell anemia, infections like malaria, etc.; however, iron deficiency is usually the main cause. Iron-deficient anemia affected 1.2 billion people in 2013 [2]. Studies reported that anemia in infancy and children may lead to irreversible body development, including the brain, which is the fastest developing organ in infancy and early childhood and increased mortality and morbidity [3,4,5]. Severe maternal anemia can contribute to adverse pregnancy outcomes and affect the iron status of newborns [6,7].

National nutrition and health surveys are not only important indicators of economic and social development, healthcare, and the population health quality of a nation or region, but also provide basic information necessary to formulate national strategies for public health and disease prevention and control. China carried out its first national nutrition and health survey in 1959, and it has subsequently been conducted every ten years since 1982 [8]. The determination of hemoglobin content is the most commonly used method for evaluating anemia status. China uses the determination of hemoglobin as a monitoring indicator of anemia in the national nutrition survey. The global prevalence of anemia in 2010 was 32.9% [1]. In 2002, the China National Nutrition and Health Survey indicated that the anemia prevalence rate of Chinese residents was 20.1%, with an urban level of 18.2% and rural level of 20.8% [9]. In 2010–2012, China completed its fourth National Nutrition and Health Survey. In this paper, we take monitoring data from the 2010–2012 China National Nutrition and Health Survey to evaluate the prevalence of anemia and the improvement status for rural residents in this decade. The hemoglobin level was determined by the cyanmethemoglobin method, which is a frequently-used method for hemoglobin determination in many anemia-related studies [10,11,12,13], and one of the methods generally recommended for use in surveys to determine the population prevalence of anemia by the World Health Organization (WHO) [14].

## 2. Subjects and Methods

### 2.1. Sample Design and Study Population

The 2010–2012 China National Nutrition and Health Survey (CNNHS) was a national, representative, and cross-sectional study on diet and chronic disease. It covered all 31 provinces, autonomous regions, and municipalities directly under the central government throughout China (with the exceptions of Taiwan, Hong Kong, and Macao). Participants were recruited by a random stratified multistage cluster sampling design. The CNNHS contained four strata: large cities, small-to-medium-sized cities, ordinary rural areas, and poor rural areas, according to their economic characteristics and social development. Large cities were municipality and provincial capitals that contained a population of more than one million people. Small-to-medium cities were defined as downtown areas that excluded the large cities. Poor rural areas were key poverty counties that were designated according to the “2001–2010 National Rural Poverty Alleviation and Development Program”. Ordinary rural areas were counties that excluded the key poverty counties. The sample size was about 160,000, which was calculated according to the low incidence of diabetes (3%), adults above 18 years old (78%), and the lost rate of follow up (10%). According to the population proportion, the first stage of sampling involved a total of 150 counties as monitoring sites, including 34 large cities, 41 small-to-medium cities, 30 ordinary rural areas, and 45 poor rural areas. The second stage involved the random selection of six village committees in each monitoring site. The third stage involved the random selection of 75 families in each village committee [15].

**Figure 1:**
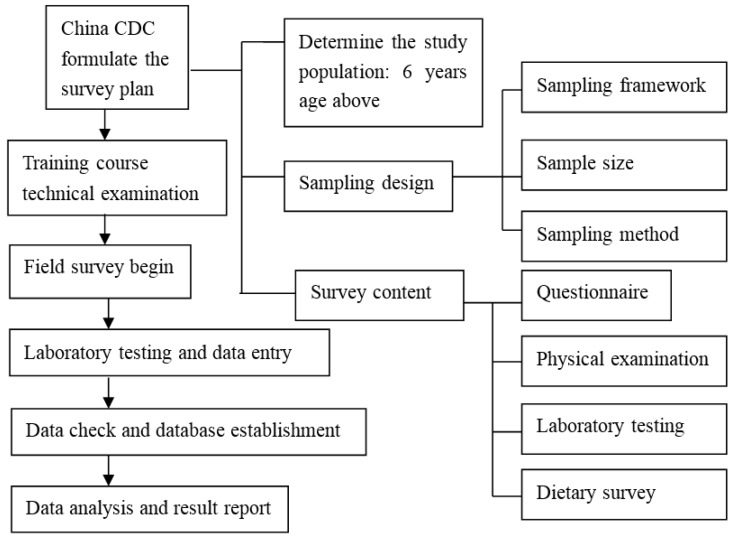
The Chinese national nutrition and health survey flow chart. CDC (Chinese Center for Disease Control and Prevention).

All procedures involving human subjects were approved by the Ethical Committee of Institute of Nutrition and Food Safety, Chinese Center for Disease Control and Prevention (2013-018). For residents under the age of 18, informed written consent was obtained from the participants’ parents prior to the start of the study. For residents above the age of 18, informed written consent was obtained from the participants. The flow chart of the National Nutrition and Health Survey was shown in Figure 1.

### 2.2. Blood Sample and Hemoglobin Measurement

Peripheral whole venous and fasting blood were first collected in EDTA (Ethylene Diamine Tetraacetic Acid) tubes, where 10 μL anticoagulant whole blood by 10 μL quantitative capillary tube (Drummond Scientific Company, Broomall, PA, USA) were collected especially for the hemoglobin test. All blood samples were used for double determination.

We used the cyanmethemoglobin method to analyze the hemoglobin level. It is the most reliable laboratory method for the quantitative determination of hemoglobin, and serves as a reference for the comparison and standardization of other methods [14]. Laboratory operations staff participated in the uniform national team training and examination. After becoming qualified, they could take part in the hemoglobin measurement. The intra- and inter-assay CV (Coefficient of Variation) in the analysis method were 1.03%–2.91% and 2.04%–3.70%, respectively. Blind samples (high and low value hemoglobin) were measured and qualified before proceeding with fieldwork. After the official start of the fieldwork, we tested one quality control analysis every thirty samples.

### 2.3. Criteria of Anemia

It is well-known that normal hemoglobin distributions vary with age, gender, and altitude. Therefore, the correct interpretation of hemoglobin requires the consideration of modulating factors in selecting appropriate cut-off values. Those values at sea level for hemoglobin corresponding to anemia are presented in Table 1 [14]. Hemoglobin levels at various altitudes are shown in Table 2 [14].

### 2.4. Data Check

The determination of hemoglobin level was finished in each monitoring site, and data were input into the unified soft “China National Nutrition and Health Survey System Platform”. Therefore, the unified data check principle was mainly formulated on the following aspects: (1) whether the individual code was repeated; (2) the difference of two parallel determination values should be less than 20% of the mean; (3) after receiving the reported data, we calculated the hemoglobin level according to the absorbance value of spectrophotometer; and finally, compared with the reported values. Thus, the calculated hemoglobin level and reported values should be the same. According to the unified data check principle, the problem record was returned to the monitoring site for the second check.

### 2.5. Statistical Analysis

We used the population figures released by the national bureau of statistics in 2009 as a standard population. Hemoglobin levels and anemia rate analyses were adjusted for sample weights and the clustered survey design [15]. Hemoglobin results were expressed as means ± standard deviation (SD). An ANOVA (Analysis of variance) test (Student–Newman–Keuls) was used to compare multiple groups of hemoglobin levels. A *t*-test was used to compare two groups of hemoglobin levels. The anemia rate was expressed as the rate, followed by the 95% CI (Confidence interval). The Rao–Scott Chi-square test was used to compare anemia prevalence among different groups. All statistical analyses were performed using Statistical Analysis Systems 9.2 software (SAS Institute Inc., Cary, NC, USA).

## 3. Results

The total sample size was originally designed to about 160,000 people, of which about 87,000 people were invited from rural areas and 80,814 people participated in a medical examination. Excluding the people who did not wish to participate and unqualified data, the rural population distribution by age, sex, and area in city and rural area in the final study is presented in Table 3. Around 73,182 subjects from the rural area were involved in the blood hemoglobin measurement, including 33,826 men (46.2%) and 39,356 women (53.8%). Additionally, 1763 pregnant women were also included.

The average hemoglobin of a Chinese rural resident was 145.92 g/L. Except for the 6–11 age group residents, the hemoglobin levels of males were higher than that of females among other age groups. The hemoglobin level of all age groups in rural residents is shown in Table 4. The average hemoglobin of rural pregnant women was 123.5 g/L. There were significant differences in hemoglobin levels between different age groups (*F* = 324.18, *p* < 0.0001). The hemoglobin of 6–11-year-old children was the lowest, and that of the 18–44-year-old adults was the highest. The hemoglobin of poor rural areas was significantly lower than that in the ordinary rural areas (*t* = 3.21, *p* = 0.0013). The female hemoglobin rate was significantly lower than that of males (*t* = 105.46, *p* < 0.0001).

The prevalence of anemia in Chinese rural residents was 9.7%, with males at 7.2% and females at 12.4%. The prevalence of anemia also appeared to increase with age, with a high prevalence of the elderly aged over 60. The prevalence of anemia in childbearing women between 18 and 44 years was higher than all other age groups. The prevalence of anemia for all age groups in rural residents is shown in Table 5. There were significant differences in anemia prevalence rate between different age groups (χ^2^ = 169.2, *p* < 0.0001). The prevalence of anemia in 6–11-year-old children was the lowest, and was highest in the elderly over 60 years old. The anemia rate in poor rural areas was significantly higher than that of ordinary rural areas (χ^2^ = 47.5, *p* < 0.0001). The female anemia rate was significantly higher than that of males (χ^2^ = 228.4, *p* < 0.0001).

For rural pregnant women, the anemia prevalence rate was 17.5% (15.8%–19.2%), among which the ordinary rural area level was 16.1% (14.0%–18.1%) and the poor rural area level was 20.0% (17.1%–23.0%).

In 2002, the anemia prevalence rate of Chinese rural residents (aged 6 years and above) was 22.2%, among which the rate in males was 18.4% and in females was 25.5%. Compared with that in 2002, the anemia rate of Chinese rural residents aged 6 years and above fell by 12.5 percentage points (χ^2^ = 3815.9, *p* < 0.0001), male levels fell by 11.2 percentage points (χ^2^ = 1453.1, *p* < 0.0001), and females fell by 13.1 percentage points (χ^2^ = 2420.3, *p* < 0.0001). The anemia rate of 6–11-year-old children fell by 7.5 percentage points (χ^2^ = 320.4, *p* < 0.0001). Teenagers aged 12–17 years fell by 9.4 percentage points (χ^2^ = 371.0, *p* < 0.0001). Rural residents in the age group 18–44 years fell by 11.8 percentage points (χ^2^ = 788.0, *p* < 0.0001), and rural residents aged 45–59 years fell by 15.4 percentage points (χ^2^ = 1559.3, *p* < 0.0001). Elderly people aged 60 years and above fell by 19.0 percentage points (χ^2^ = 1360.9, *p* < 0.0001). The anemia prevalence rate of all age groups in rural residents in the 2002 survey is shown in Table 6. The anemia prevalence rate of rural pregnant women in the 2002 survey was 30.4%, compared with that in 2002, the rate of anemia in rural pregnant women fell by 12.9 percentage points (χ^2^ = 129.7, *p* < 0.0001).

## 4. Discussion and Conclusions

Anemia is a global public health problem affecting both developing and developed countries, and is always used as an indicator of both poor nutrition and poor health. In this survey, the blood hemoglobin level of the Chinese rural area population was 145.92 ± 0.83 g/L. The anemia prevalence of the Chinese rural area population was 9.7% (95% CI: 9.4%–10.0%), which was lower than the global anemia prevalence (32.9%) in 2010 [1].

The prevalence of anemia in Chinese rural residents is significantly lower than it was ten years ago. The prevalence of anemia in 18–44-year-old women is much improved, but is still the highest in all age groups, even higher than that of women over 60 years of age. This may be related to the 18–44-year-old group being involved in periods of birth, breast-feeding, and menstruation. In this period, the body requires a lot of iron, otherwise it results in iron deficiency anemia (IDA), which develops when body stores of iron drop too low to support normal red blood cell (RBC) production. The WHO has conducted a study on the anemic status of childbearing-aged women from 107 countries all over the world and found that the anemia rate of childbearing aged women fell from 33% in 1995 to 29% in 2011 [16]. A Japanese study on the prevalence of anemia in healthy Japanese women also showed that women aged 20 to 49 had a significantly higher rate of anemia than other age groups of women [17]. The problems of anemia in childbearing age women caused wide attention around the world; indeed, The World Health Assembly in 2014 (WHA) proposed global nutrition goals which aim at reducing anemia in women of childbearing age by 50% before 2025 [18].

In addition, the prevalence of anemia in the elderly is higher than the national average, which is equivalent to that in developed countries. It has been reported that in the United States, more than 10% have IDA at age 65 and older [19]. Anemia is common in the elderly, and its impact on various important health outcomes has recently been clearly demonstrated [20,21,22]—especially for the very elderly. In the very elderly (age 85 and older), IDA carries an increased risk of mortality (hazard ratio 1.41) in addition to conditions causing anemia [23]. China has entered the aging society, and in 2014, the elderly population reached 200 million. In the next 20 years, the elderly population will enter a rapid growth period and reach a third of the population in 2050. Therefore, more attention needs to be directed towards the intervention of anemia in the elderly, and actions need to be considered to improve the quality of life for the elderly.

Iron deficiency is the most important cause of anemia. In 2013, anemia due to iron deficiency resulted in about 183,000 deaths, down from 213,000 deaths in 1990 [2]. The total dietary iron intake of rural adults above 18 years old was 21.2 mg/day [24], down from 23.1 mg in the 2002 survey. Iron absorption has obvious differences in different food types. For example, iron absorption is one percent for rice, three percent for corn, five percent for flour, and 22% for animal meat [25]. In this survey, pork and poultry intake were 59.9 g/day and 13.1 g/day, respectively [24], increased from 47.2 g/day and 10.6 g/day in 2002. Therefore, we believe that the increase of meat consumption may help to improve anemia levels. One study on the iron status of the Chinese population in poor rural areas (*n* = 4186) [26] found that the average concentration of serum ferritin (SF) for residents in poor rural areas was 98.7 ng/mL—140 ng/mL for males, and 72.1 ng/mL for females. The average serum transferrin receptor (sTfR) concentration for the anemic population in poor rural areas was 4.05 mg/L—3.48 mg/L for anemic males, and 4.47 mg/L for anemic females [26]. Furthermore, the iron deficiency rate of anemic residents in poor rural areas was 32.6%, 15.5% for anemic males and 43.7% for anemic females [26]. Criteria of sTfR >5.0 mg/L (male) and sTfR >4.4 mg/L (female) or SF <15 ng/mL can be diagnosed as iron deficiency. As there were no related iron status data in the 2002 nutritional survey, a conclusion could not be made that the decline of iron deficiency anemia was the main cause of anemia improvement, even though it is generally believed that iron-deficiency is the main cause of anemia. Public health strategies for the prevention and improvement of anemia levels include improving dietary diversity, food iron and folic acid fortification, and increases in the intake of iron nutritional supplements. Food fortification is the most common method in which to supplement nutrients in food, and could effectively decrease the incidence of nutrient deficiencies [27]. This is demonstrated by Europe and the United States, where iron fortification in flour has improved the iron anemia prevalence rate from 30% to 10% [1]. In China, soy sauce fortified with NaFeEDTA (sodium iron ethylene diamine tetraacetic acid) has been used to improve the anemia situation. It has been reported that NaFeEDTA-fortified soy sauce has had an obvious effect on correcting iron deficiency and reducing anemia [28,29]. In addition to food fortification, we also concluded that the following factors might also be conducive to changes in anemia prevalence. First, actively promoting the risks of anemia through school education, the media, and community health programs. Second, by improving the living standards of the residents, especially in dietary diversity and an increased intake of iron-rich foods. Third, the increase of public health awareness, regular physical examinations, and adopting intervention measures after the discovery of anemia.

A variety of reasons have contributed to the decrease of the prevalence of anemia. There are some limitations to this study; as we initially did not think that there had been great improvements in the prevalence of anemia, the nutrition survey did not include examining some of the causes of improvement in anemia rates, such as iron supplements, NaFeEDTA-fortified soy sauce usage, or other iron-fortified food. The strength of the study lies in the fact that the data used comes directly from the CNNHS, which covers all 31 provinces, autonomous regions, and municipalities directly under the central government throughout China, therefore providing this study with very good representativeness and an accurate assessment on the status of anemia.

In short, the prevalence of anemia in Chinese rural residents has improved greatly compared with that ten years ago. Future attention needs to be paid to the evaluation of the intervention measures used to improve anemia, strengthen the concern for key crowd anemia, and put forward effective and safe measures for anemia improvement in order to achieve the nutritional disease control target proposed by the development of Chinese Food and Nutrition Program (2014–2030), which aims at an anemia prevalence rate of under 10% for the whole population.

## Figures and Tables

**Table 1:** Hemoglobin cutoffs used to define anemia in people living at sea level.

Age or Sex Group	Hemoglobin (g/L)
Children 6 months to 59 months	110
Children 5–11 years	115
Children 12–14 years	120
Non-pregnant women (above 15 years of age)	120
Pregnant women	110
Men (above 15 years of age)	130

**Table 2:** Normal increases of hemoglobin values related to long-term altitude exposure.

Altitude (m)	Increase in Hemoglobin (g/L)
<1000	0
1000	+2
1500	+5
2000	+8
2500	+13
3000	+19
3500	+27
4000	+35
4500	+45

**Table 3:** Study population distribution by age, sex, and type of rural area (*n*).

Age (Years)	Ordinary Rural Area			Poor Rural Area			Total		
	Male	Female	Total	Male	Female	Total	Male	Female	Total
6–11	2755	2618	5373	1487	1375	2862	4242	3993	8235
12–17	2597	2531	5128	1515	1549	3064	4112	4080	8192
18–44	4787	6666	11,453	3409	4507	7916	8196	11,173	19,369
45–59	6214	7938	14,152	3271	4064	7335	9485	12,002	21,487
≥60	5235	5468	10,703	2556	2640	5196	7791	8108	15,899
Total	21,588	25,221	46,809	12,238	14,135	26,373	33,826	39,356	73,182

**Table 4:** 2010–2012 China’s rural residents’ hemoglobin levels (means ± standard deviation (SD), g/L).

Age (Year)	Ordinary Rural Area			Poor Rural Area			Total		
	Male	Female	Total	Male	Female	Total	Male	Female	Total
6–11	137.02 ± 0.94	137.40 ± 0.98	137.20 ± 0.93	136.78 ± 1.36	136.52 ± 1.35	136.65 ± 1.33	136.95 ± 0.77	137.12 ± 0.79	137.03 ± 0.76
12–17	151.30 ± 1.19	139.96 ± 1.02	146.09 ± 1.06	146.14 ± 1.57	138.20 ± 1.10	142.45 ± 1.33	149.72 ± 0.97	139.41 ± 0.78	144.96 ± 0.85
18–44	159.02 ± 1.42	136.12 ± 1.19	148.14 ± 1.26	156.29 ± 1.56	137.57 ± 1.52	147.42 ± 1.32	158.22 ± 1.12	136.54 ± 0.95	147.93 ± 0.98
45–59	155.34 ± 1.11	138.14 ± 1.18	146.88 ± 1.13	153.17 ± 1.45	138.83 ± 2.11	146.18 ± 1.57	154.74 ± 0.90	138.33 ± 1.03	146.69 ± 0.93
≥60	150.28 ± 1.34	137.70 ± 1.21	143.87 ± 1.25	148.32 ± 1.60	137.10 ± 2.11	142.65 ± 1.79	149.72 ± 1.10	137.53 ± 1.07	143.52 ± 1.05
Total	154.63 ± 1.18	137.21 ± 1.03	146.25 ± 1.04	151.94 ± 1.38	137.72 ± 1.51	145.12 ± 1.25	153.85 ± 0.94	137.40 ± 0.85	145.92 ± 0.83

Note: Pregnant women were excluded.

**Table 5:** 2010–2012 China’s rural residents’ anemia prevalence (% 95% CI (Confidence interval)).

Age (Years)	Ordinary Rural Area			Poor Rural Area			Total		
	Male	Female	Total	Male	Female	Total	Male	Female	Total
6–11	4.6 (3.8–5.4)	5.5 (4.6–6.4)	5.0 (4.4–5.6)	7.0 (5.7–8.3)	6.4 (5.1–7.7)	6.7 (5.8–7.6)	5.3 (4.6–6.0)	5.8 (5.0–6.5)	5.5 (5.0–6.0)
12–17	5.4 (4.4–6.3)	7.6 (6.6–8.7)	6.4 (5.7–7.1)	12.2 (10.5–14.0)	11.4 (9.7–13.1)	11.8 (10.6–13.1)	7.5 (6.6–8.3)	8.8 (7.9–9.7)	8.1 (7.5–8.7)
18–44	5.0 (4.2–5.8)	14.4 (13.2–15.6)	9.5 (8.7–10.2)	8.4 (7.0–9.9)	14.7 (13.1–16.4)	11.4 (10.3–12.5)	6.0 (5.3–6.7)	14.5 (13.5–15.4)	10.0 (9.4–10.6)
45–59	6.6 (5.8–7.4)	11.3 (10.3–12.4)	8.9 (8.3–9.6)	9.9 (8.5–11.4)	12.8 (11.3–14.4)	11.4 (10.3–12.4)	7.5 (6.8–8.2)	11.7 (10.9–12.6)	9.6 (9.0–10.1)
≥60	11.9 (10.6–13.1)	11.3 (10.2–12.4)	11.6 (10.7–12.4)	15.7 (13.7–17.7)	14.5 (12.5–16.6)	15.1 (13.7–16.5)	12.9 (11.9–14.0)	12.2 (11.2–13.2)	12.6 (11.9–13.3)
Total	6.2 (5.7–6.6)	12.0 (11.4–12.7)	9.0 (8.6–9.4)	9.9 (9.0–10.7)	13.3 (12.4–14.2)	11.5 (10.9–12.1)	7.2 (6.8–7.7)	12.4 (11.9–12.9)	9.7 (9.4–10.0)

Note: Pregnant women were excluded.

**Table 6:** Comparison of anemia prevalence rates of six years and older Chinese rural residents between 2002 and 2012 (%).

Age (Years)	2002			2012		
	Male	Female	Total	Male	Female	Total
6–11	13.1	12.9	13.0	5.3	5.8	5.5
12–17	16.2	19.0	17.5	7.5	8.8	8.1
18–44	14.6	27.2	21.8	6.0	14.5	10.0
45–59	21.5	28.0	25.0	7.5	11.7	9.6
≥60	31.8	31.3	31.6	12.9	12.2	12.6
Total	18.4	25.5	22.2	7.2	12.4	9.7

Note: Pregnant women were excluded.
